# Supplementary material for: Feature selection with vector-symbolic architectures: a case study on microbial profiles of shotgun metagenomic samples of colorectal cancer
Source: Brief Bioinform. 2025 Apr 24;26(2):bbaf177. doi: 10.1093/bib/bbaf177 (PMC12018301; doi:10.1093/bib/bbaf177)
Supplement: Supplementary_Table_S1_bbaf177 [file supplementary_table_s1_bbaf177.docx]

**Feature selection with vector-symbolic architectures: a case study on microbial profiles of shotgun metagenomic samples of colorectal cancer**

Fabio Cumbo^1^, Simone Truglia^2^, Emanuel Weitschek^2^, Daniel Blankenberg^1,3,*^

^1^ Center for Computational Life Sciences, Lerner Research Institute, Cleveland Clinic, Cleveland, OH, USA

^2^ Department of Engineering, Uninettuno University, Rome, Italy

^3^ Department of Molecular Medicine, Cleveland Clinic Lerner College of Medicine, Case Western Reserve University, Cleveland, OH, USA

^*^ To whom correspondence should be addressed. Email: [blanked2@ccf.org](mailto:blanked2@ccf.org)

Supplementary Table S1

|  |  | **Binarized validation datasets** | | | | |
| --- | --- | --- | --- | --- | --- | --- |
|  |  | **Unstratified** | **w/ male only** | **w/ female only** | **w/ adult only** | **w/ senior only** |
| *chopin2*  (powered by *hdlib*) | *Accuracy* | 60.86% | 59.12% | 65.09% | 60.92% | 61.16% |
|  | *Precision* | 61.58% | 61.84% | 71.32% | 65.14% | 68.42% |
|  | *Recall* | 60.86% | 59.12% | 65.09% | 60.92% | 61.16% |
|  | *F1* | 60.39% | 58.33% | 60.60% | 58.32% | 57.02% |

| **Table S1:** Performance metrics (accuracy, precision, recall, and F1 score) on the validation HD-based model (*YachidaS_2019*) built on the binarized dataset with the set of selected species only. |
| --- |
