# Supplementary material for: Feature selection with vector-symbolic architectures: a case study on microbial profiles of shotgun metagenomic samples of colorectal cancer
Source: Brief Bioinform. 2025 Apr 24;26(2):bbaf177. doi: 10.1093/bib/bbaf177 (PMC12018301; doi:10.1093/bib/bbaf177)
Supplement: Supplementary_Table_S2_bbaf177 [file supplementary_table_s2_bbaf177.docx]

**Feature selection with vector-symbolic architectures: a case study on microbial profiles of shotgun metagenomic samples of colorectal cancer**

Fabio Cumbo^1^, Simone Truglia^2^, Emanuel Weitschek^2^, Daniel Blankenberg^1,3,*^

^1^ Center for Computational Life Sciences, Lerner Research Institute, Cleveland Clinic, Cleveland, OH, USA

^2^ Department of Engineering, Uninettuno University, Rome, Italy

^3^ Department of Molecular Medicine, Cleveland Clinic Lerner College of Medicine, Case Western Reserve University, Cleveland, OH, USA

^*^ To whom correspondence should be addressed. Email: [blanked2@ccf.org](mailto:blanked2@ccf.org)

Supplementary Table S2

|  |  | **Binarized datasets** | | | | |
| --- | --- | --- | --- | --- | --- | --- |
|  |  | **Unstratified** | **w/ male only** | **w/ female only** | **w/ adult only** | **w/ senior only** |
| *chopin2*  (powered by *hdlib*) | *Accuracy* | 81.31% | 78.33% | 79.43% | 84.33% | 82.28% |
|  | *Precision* | 75.84% | 78.62% | 83.29% | 90.21% | 85.08% |
|  | *Recall* | 75.11% | 76.67% | 82.29% | 89.87% | 83.38% |
|  | *F1* | 74.94% | 76.40% | 82.21% | 89.85% | 83.06% |
| Random Forest | *Accuracy* | 80.30% | 83.33% | 78.09% | 89.00% | 77.50% |
|  | *Precision* | 92.01% | 87.85% | 97.14% | 98.18% | 92.66% |
|  | *Recall* | 84.16% | 97.41% | 73.80% | 90.11% | 75.68% |
|  | *F1* | 80.18% | 86.14% | 74.42% | 87.99% | 72.86% |
| Decision Tree | *Accuracy* | 72.56% | 65.83% | 76.57% | 80.77% | 72.57% |
|  | *Precision* | 90.00% | 75.89% | 96.00% | 96.66% | 85.77% |
|  | *Recall* | 71.61% | 70.00% | 65.42% | 75.44% | 69.95% |
|  | *F1* | 70.24% | 68.68% | 66.48% | 78.61% | 64.96% |
| SVM | *Accuracy* | 77.70% | 81.66% | 76.57% | 80.64% | 75.00% |
|  | *Precision* | 82.54% | 86.53% | 89.28% | 97.77% | 83.50% |
|  | *Recall* | 87.79% | 90.00% | 78.28% | 86.75% | 75.14% |
|  | *F1* | 78.47% | 84.27% | 72.80% | 82.17% | 69.86% |
| Logistic  Regression | *Accuracy* | 81.86% | 80.00% | 78.00% | 87.18% | 73.82% |
|  | *Precision* | 89.38% | 83.28% | 90.95% | 98.18% | 77.19% |
|  | *Recall* | 88.01% | 88.75% | 74.28% | 84.53% | 71.36% |
|  | *F1* | 82.18% | 82.86% | 73.90% | 87.60% | 69.20% |
| XGBoost | *Accuracy* | 79.79% | 76.66% | 80.85% | 84.37% | 70.07% |
|  | *Precision* | 85.43% | 85.47% | 96.00% | 89.66% | 76.33% |
|  | *Recall* | 83.80% | 84.75% | 70.76% | 82.55% | 77.77% |
|  | *F1* | 82.52% | 77.71% | 71.25% | 80.18% | 69.78% |
| Neural Network | *Accuracy* | 83.94% | 83.33% | 74.19% | 79.87% | 72.57% |
|  | *Precision* | 87.13% | 84.12% | 75.16% | 77.61% | 74.82% |
|  | *Recall* | 93.48% | 100.00% | 81.80% | 96.25% | 86.95% |
|  | *F1* | 84.12% | 85.52% | 72.16% | 81.80% | 72.00% |

| **Table S2:** Comparison of the HD-based model performance versus the selected classical approaches based on the accuracy, precision, recall, and F1 scores, considering models with method-specific selected features only. Models are built over the binarized datasets. |
| --- |
