# Supplementary material for: Feature selection with vector-symbolic architectures: a case study on microbial profiles of shotgun metagenomic samples of colorectal cancer
Source: Brief Bioinform. 2025 Apr 24;26(2):bbaf177. doi: 10.1093/bib/bbaf177 (PMC12018301; doi:10.1093/bib/bbaf177)
Supplement: Supplementary_Table_S3_bbaf177 [file supplementary_table_s3_bbaf177.docx]

**Feature selection with vector-symbolic architectures: a case study on microbial profiles of shotgun metagenomic samples of colorectal cancer**

Fabio Cumbo^1^, Simone Truglia^2^, Emanuel Weitschek^2^, Daniel Blankenberg^1,3,*^

^1^ Center for Computational Life Sciences, Lerner Research Institute, Cleveland Clinic, Cleveland, OH, USA

^2^ Department of Engineering, Uninettuno University, Rome, Italy

^3^ Department of Molecular Medicine, Cleveland Clinic Lerner College of Medicine, Case Western Reserve University, Cleveland, OH, USA

^*^ To whom correspondence should be addressed. Email: [blanked2@ccf.org](mailto:blanked2@ccf.org)

Supplementary Table S3

|  |  | **Relative Abundance (RA) datasets** | | | | |
| --- | --- | --- | --- | --- | --- | --- |
|  |  | **Unstratified** | **w/ male only** | **w/ female only** | **w/ adult only** | **w/ senior only** |
| *chopin2*  (powered by *hdlib*) | *Accuracy* | 70.50% | 74.17% | 69.71% | 73.51% | 72.57% |
|  | *Precision* | 68.18% | 74.18% | 50.70% | 71.29% | 78.82% |
|  | *Recall* | 67.44% | 73.33% | 52.19% | 70.74% | 77.28% |
|  | *F1* | 67.20% | 73.30% | 50.11% | 70.22% | 76.94% |
| Random Forest | *Accuracy* | 77.80% | 79.16% | 82.19% | 85.36% | 79.70% |
|  | *Precision* | 90.63% | 84.75% | 89.33% | 98.18% | 83.73% |
|  | *Recall* | 86.24% | 87.41% | 83.14% | 92.21% | 85.00% |
|  | *F1* | 78.81% | 81.98% | 79.85% | 84.18% | 78.14% |
| Decision Tree | *Accuracy* | 70.02% | 69.16% | 71.23% | 72.51% | 66.61% |
|  | *Precision* | 88.68% | 80.81% | 100.00% | 94.18% | 68.76% |
|  | *Recall* | 75.45% | 76.91% | 62.95% | 69.47% | 69.18% |
|  | *F1* | 71.37% | 72.16% | 61.99% | 72.37% | 64.55% |
| SVM | *Accuracy* | 67.85% | 70.83% | 74.00% | 66.06% | 77.35% |
|  | *Precision* | 72.77% | 76.01% | 87.77% | 76.14% | 84.54% |
|  | *Recall* | 93.66% | 90.61% | 77.14% | 83.86% | 94.00% |
|  | *F1* | 70.86% | 75.69% | 69.97% | 67.38% | 75.66% |
| Logistic  Regression | *Accuracy* | 66.31% | 71.66% | 65.90% | 64.19% | 73.75% |
|  | *Precision* | 70.98% | 77.53% | 75.00% | 70.46% | 88.88% |
|  | *Recall* | 85.86% | 91.08% | 75.14% | 78.67% | 89.68% |
|  | *F1* | 68.63% | 73.67% | 62.83% | 67.80% | 73.78% |
| XGBoost | *Accuracy* | 76.24% | 73.33% | 78.00% | 76.27% | 73.75% |
|  | *Precision* | 92.88% | 83.84% | 100.00% | 86.36% | 77.78% |
|  | *Recall* | 82.30% | 87.83% | 72.95% | 83.68% | 86.27% |
|  | *F1* | 77.68% | 76.68% | 70.38% | 76.36% | 75.52% |
| Neural Network | *Accuracy* | 66.85% | 73.33% | 65.80% | 72.51% | 73.75% |
|  | *Precision* | 69.79% | 77.65% | 66.28% | 77.50% | 73.77% |
|  | *Recall* | 76.80% | 90.41% | 81.14% | 82.43% | 92.50% |
|  | *F1* | 68.81% | 77.21% | 66.16% | 74.80% | 74.42% |

| **Table S3:** Comparison of the HD-based model performance versus the selected classical approaches based on the accuracy, precision, recall, and F1 scores, considering models with method-specific selected features only. Models are built over the original datasets with relative abundance (RA) profiles. |
| --- |
