# Supplementary material for: Feature selection with vector-symbolic architectures: a case study on microbial profiles of shotgun metagenomic samples of colorectal cancer
Source: Brief Bioinform. 2025 Apr 24;26(2):bbaf177. doi: 10.1093/bib/bbaf177 (PMC12018301; doi:10.1093/bib/bbaf177)
Supplement: Supplementary_Table_S4_bbaf177 [file supplementary_table_s4_bbaf177.docx]

**Feature selection with vector-symbolic architectures: a case study on microbial profiles of shotgun metagenomic samples of colorectal cancer**

Fabio Cumbo^1^, Simone Truglia^2^, Emanuel Weitschek^2^, Daniel Blankenberg^1,3,*^

^1^ Center for Computational Life Sciences, Lerner Research Institute, Cleveland Clinic, Cleveland, OH, USA

^2^ Department of Engineering, Uninettuno University, Rome, Italy

^3^ Department of Molecular Medicine, Cleveland Clinic Lerner College of Medicine, Case Western Reserve University, Cleveland, OH, USA

^*^ To whom correspondence should be addressed. Email: [blanked2@ccf.org](mailto:blanked2@ccf.org)

Supplementary Table S4

|  | **Binarized datasets – Wilcoxon rank-sum test (one-sided, unpaired)** | | | | |
| --- | --- | --- | --- | --- | --- |
| *chopin2* vs | **Unstratified** | **w/ male only** | **w/ female only** | **w/ adult only** | **w/ senior only** |
| Random Forest | 0.2996 | 0.1217 | 0.7711 | 0.5000 | 0.8828 |
| Decision Tree | 0.8452 | 0.9547 | 0.8286 | 0.9674 | 0.9839 |
| SVM | 0.5830 | 0.1951 | 0.9207 | 0.9904 | 0.9839 |
| Logistic Regression | 0.1043 | 0.3342 | 0.9201 | 0.6648 | 0.9634 |
| XGBoost | 0.2738 | 0.5885 | 0.7021 | 0.9331 | 0.9839 |
| Neural Network | 0.0374 | 0.1691 | 0.9160 | 0.9875 | 0.9442 |

| **Table S4:** P-values as result from the Wilcoxon rank-sum test (one-sided, unpaired) based on the accuracy scores observed in the 5-folds produced during the cross-validation of our model and the 6 classical models over the 5 binarized datasets. It shows no statistical significance except for the comparison with the Neural Network in the context of the Unstratified dataset, with a p-value <0.05 (in green). |
| --- |
