# Supplementary material for: Feature selection with vector-symbolic architectures: a case study on microbial profiles of shotgun metagenomic samples of colorectal cancer
Source: Brief Bioinform. 2025 Apr 24;26(2):bbaf177. doi: 10.1093/bib/bbaf177 (PMC12018301; doi:10.1093/bib/bbaf177)
Supplement: SupplementaryMaterials_bbaf177 [file supplementarymaterials_bbaf177.docx]

# SUPPLEMENTARY MATERIALS

**Feature selection with vector-symbolic architectures: a case study on microbial profiles of shotgun metagenomic samples of colorectal cancer**

Fabio Cumbo^1^, Simone Truglia^2^, Emanuel Weitschek^2^, Daniel Blankenberg^1,3,*^

^1^ Center for Computational Life Sciences, Lerner Research Institute, Cleveland Clinic, Cleveland, OH, USA

^2^ Department of Engineering, Uninettuno University, Rome, Italy

^3^ Department of Molecular Medicine, Cleveland Clinic Lerner College of Medicine, Case Western Reserve University, Cleveland, OH, USA

^*^ To whom correspondence should be addressed. Email: [blanked2@ccf.org](mailto:blanked2@ccf.org)

|  |  | **Binarized validation datasets** | | | | |
| --- | --- | --- | --- | --- | --- | --- |
|  |  | **Unstratified** | **w/ male only** | **w/ female only** | **w/ adult only** | **w/ senior only** |
| *chopin2*  (powered by *hdlib*) | *Accuracy* | 60.86% | 59.12% | 65.09% | 60.92% | 61.16% |
|  | *Precision* | 61.58% | 61.84% | 71.32% | 65.14% | 68.42% |
|  | *Recall* | 60.86% | 59.12% | 65.09% | 60.92% | 61.16% |
|  | *F1* | 60.39% | 58.33% | 60.60% | 58.32% | 57.02% |

| **Table S1:** Performance metrics (accuracy, precision, recall, and F1 score) on the validation HD-based model (*YachidaS_2019*) built on the binarized dataset with the set of selected species only. |
| --- |

|  |  | **Binarized datasets** | | | | |
| --- | --- | --- | --- | --- | --- | --- |
|  |  | **Unstratified** | **w/ male only** | **w/ female only** | **w/ adult only** | **w/ senior only** |
| *chopin2*  (powered by *hdlib*) | *Accuracy* | 81.31% | 78.33% | 79.43% | 84.33% | 82.28% |
|  | *Precision* | 75.84% | 78.62% | 83.29% | 90.21% | 85.08% |
|  | *Recall* | 75.11% | 76.67% | 82.29% | 89.87% | 83.38% |
|  | *F1* | 74.94% | 76.40% | 82.21% | 89.85% | 83.06% |
| Random Forest | *Accuracy* | 80.30% | 83.33% | 78.09% | 89.00% | 77.50% |
|  | *Precision* | 92.01% | 87.85% | 97.14% | 98.18% | 92.66% |
|  | *Recall* | 84.16% | 97.41% | 73.80% | 90.11% | 75.68% |
|  | *F1* | 80.18% | 86.14% | 74.42% | 87.99% | 72.86% |
| Decision Tree | *Accuracy* | 72.56% | 65.83% | 76.57% | 80.77% | 72.57% |
|  | *Precision* | 90.00% | 75.89% | 96.00% | 96.66% | 85.77% |
|  | *Recall* | 71.61% | 70.00% | 65.42% | 75.44% | 69.95% |
|  | *F1* | 70.24% | 68.68% | 66.48% | 78.61% | 64.96% |
| SVM | *Accuracy* | 77.70% | 81.66% | 76.57% | 80.64% | 75.00% |
|  | *Precision* | 82.54% | 86.53% | 89.28% | 97.77% | 83.50% |
|  | *Recall* | 87.79% | 90.00% | 78.28% | 86.75% | 75.14% |
|  | *F1* | 78.47% | 84.27% | 72.80% | 82.17% | 69.86% |
| Logistic  Regression | *Accuracy* | 81.86% | 80.00% | 78.00% | 87.18% | 73.82% |
|  | *Precision* | 89.38% | 83.28% | 90.95% | 98.18% | 77.19% |
|  | *Recall* | 88.01% | 88.75% | 74.28% | 84.53% | 71.36% |
|  | *F1* | 82.18% | 82.86% | 73.90% | 87.60% | 69.20% |
| XGBoost | *Accuracy* | 79.79% | 76.66% | 80.85% | 84.37% | 70.07% |
|  | *Precision* | 85.43% | 85.47% | 96.00% | 89.66% | 76.33% |
|  | *Recall* | 83.80% | 84.75% | 70.76% | 82.55% | 77.77% |
|  | *F1* | 82.52% | 77.71% | 71.25% | 80.18% | 69.78% |
| Neural Network | *Accuracy* | 83.94% | 83.33% | 74.19% | 79.87% | 72.57% |
|  | *Precision* | 87.13% | 84.12% | 75.16% | 77.61% | 74.82% |
|  | *Recall* | 93.48% | 100.00% | 81.80% | 96.25% | 86.95% |
|  | *F1* | 84.12% | 85.52% | 72.16% | 81.80% | 72.00% |

| **Table S2:** Comparison of the HD-based model performance versus the selected classical approaches based on the accuracy, precision, recall, and F1 scores, considering models with method-specific selected features only. Models are built over the binarized datasets. |
| --- |

|  |  | **Relative Abundance (RA) datasets** | | | | |
| --- | --- | --- | --- | --- | --- | --- |
|  |  | **Unstratified** | **w/ male only** | **w/ female only** | **w/ adult only** | **w/ senior only** |
| *chopin2*  (powered by *hdlib*) | *Accuracy* | 70.50% | 74.17% | 69.71% | 73.51% | 72.57% |
|  | *Precision* | 68.18% | 74.18% | 50.70% | 71.29% | 78.82% |
|  | *Recall* | 67.44% | 73.33% | 52.19% | 70.74% | 77.28% |
|  | *F1* | 67.20% | 73.30% | 50.11% | 70.22% | 76.94% |
| Random Forest | *Accuracy* | 77.80% | 79.16% | 82.19% | 85.36% | 79.70% |
|  | *Precision* | 90.63% | 84.75% | 89.33% | 98.18% | 83.73% |
|  | *Recall* | 86.24% | 87.41% | 83.14% | 92.21% | 85.00% |
|  | *F1* | 78.81% | 81.98% | 79.85% | 84.18% | 78.14% |
| Decision Tree | *Accuracy* | 70.02% | 69.16% | 71.23% | 72.51% | 66.61% |
|  | *Precision* | 88.68% | 80.81% | 100.00% | 94.18% | 68.76% |
|  | *Recall* | 75.45% | 76.91% | 62.95% | 69.47% | 69.18% |
|  | *F1* | 71.37% | 72.16% | 61.99% | 72.37% | 64.55% |
| SVM | *Accuracy* | 67.85% | 70.83% | 74.00% | 66.06% | 77.35% |
|  | *Precision* | 72.77% | 76.01% | 87.77% | 76.14% | 84.54% |
|  | *Recall* | 93.66% | 90.61% | 77.14% | 83.86% | 94.00% |
|  | *F1* | 70.86% | 75.69% | 69.97% | 67.38% | 75.66% |
| Logistic  Regression | *Accuracy* | 66.31% | 71.66% | 65.90% | 64.19% | 73.75% |
|  | *Precision* | 70.98% | 77.53% | 75.00% | 70.46% | 88.88% |
|  | *Recall* | 85.86% | 91.08% | 75.14% | 78.67% | 89.68% |
|  | *F1* | 68.63% | 73.67% | 62.83% | 67.80% | 73.78% |
| XGBoost | *Accuracy* | 76.24% | 73.33% | 78.00% | 76.27% | 73.75% |
|  | *Precision* | 92.88% | 83.84% | 100.00% | 86.36% | 77.78% |
|  | *Recall* | 82.30% | 87.83% | 72.95% | 83.68% | 86.27% |
|  | *F1* | 77.68% | 76.68% | 70.38% | 76.36% | 75.52% |
| Neural Network | *Accuracy* | 66.85% | 73.33% | 65.80% | 72.51% | 73.75% |
|  | *Precision* | 69.79% | 77.65% | 66.28% | 77.50% | 73.77% |
|  | *Recall* | 76.80% | 90.41% | 81.14% | 82.43% | 92.50% |
|  | *F1* | 68.81% | 77.21% | 66.16% | 74.80% | 74.42% |

| **Table S3:** Comparison of the HD-based model performance versus the selected classical approaches based on the accuracy, precision, recall, and F1 scores, considering models with method-specific selected features only. Models are built over the original datasets with relative abundance (RA) profiles. |
| --- |

|  | **Binarized datasets – Wilcoxon rank-sum test (one-sided, unpaired)** | | | | |
| --- | --- | --- | --- | --- | --- |
| *chopin2* vs | **Unstratified** | **w/ male only** | **w/ female only** | **w/ adult only** | **w/ senior only** |
| Random Forest | 0.2996 | 0.1217 | 0.7711 | 0.5000 | 0.8828 |
| Decision Tree | 0.8452 | 0.9547 | 0.8286 | 0.9674 | 0.9839 |
| SVM | 0.5830 | 0.1951 | 0.9207 | 0.9904 | 0.9839 |
| Logistic Regression | 0.1043 | 0.3342 | 0.9201 | 0.6648 | 0.9634 |
| XGBoost | 0.2738 | 0.5885 | 0.7021 | 0.9331 | 0.9839 |
| Neural Network | 0.0374 | 0.1691 | 0.9160 | 0.9875 | 0.9442 |

| **Table S4:** P-values as result from the Wilcoxon rank-sum test (one-sided, unpaired) based on the accuracy scores observed in the 5-folds produced during the cross-validation of our model and the 6 classical models over the 5 binarized datasets. It shows no statistical significance except for the comparison with the Neural Network in the context of the Unstratified dataset, with a p-value <0.05 (in green). |
| --- |
